# Séroprévalence et facteurs associés à l’acceptation du Conseil et Dépistage Volontaire du VIH chez l’enfant à Lubumbashi, République Démocratique du Congo

**DOI:** 10.11604/pamj.2017.28.82.9566

**Published:** 2017-09-27

**Authors:** Dieudonné Tshikwej Ngwej, Olivier Mukuku, Françoise Kaj Malonga, Oscar Numbi Luboya, Jean-Baptiste Sakatolo Kakoma, Stanis Okitotsho Wembonyama

**Affiliations:** 1 Département de Pédiatrie, Faculté de Médecine, Université de Lubumbashi, République Démocratique du Congo; 2 Bureau Provincial de ICAP/RDC, Lubumbashi, République Démocratique du Congo; 3 Ecole de Santé Publique, Université de Lubumbashi, République Démocratique du Congo; 4 Département de Gynécologie-Obstétrique, Faculté de Médecine, Université de Lubumbashi, République Démocratique du Congo

**Keywords:** VIH, enfant, séroprévalence, conseil et dépistage volontaire, acceptation, Lubumbashi, HIV, child, seroprevalence, voluntary counselling and testing, acceptance, Lubumbashi

## Abstract

**Introduction:**

Malgré le dépistage du VIH proposé lors de la naissance ou au cours des consultations préscolaires, la proportion des enfants qui croissent ou décèdent sous statut sérologique au VIH inconnu est importante en République Démocratique du Congo (RDC). L’objectif de cette étude était de déterminer la séroprévalence au cours d’un dépistage volontaire et d’identifier les facteurs associés à l’acceptation du conseil et dépistage du VIH (CDV) en dehors de la maladie ou de toute exposition au VIH dans une population pédiatrique à Lubumbashi, RDC.

**Méthodes:**

Il s’agissait d’une étude prospective transversale à visée analytique menée du 1^er^ août 2006 au 31 septembre 2007. Elle avait été réalisée dans 4 centres communautaires de CDV répartis dans 4 zones de santé de la ville de Lubumbashi en RDC (Lubumbashi, Ruashi, Kampemba et de Kenya). L’étude avait consisté à faire le dépistage volontaire du VIH chez les enfants de moins de 15 ans. Les caractéristiques sociodémographiques et les paramètres relatifs au conseil et dépistage volontaire ont été étudiés. Les analyses statistiques descriptives usuelles et une régression logistique ont été réalisées.

**Résultats:**

Sur 463 enfants dépistés du VIH, 41 (8,9%; IC 95%: 6,5%-11,9%) ont été testés positifs. L’acceptation du conseil et dépistage volontaire du VIH en dehors de la maladie ou de l’exposition au VIH était significativement plus élevée lorsque l’enfant était âgé de plus de 2 ans (Odds ratio ajusté (ORa) = 3,6 [IC 95%: 1,1-12,2]), lorsque le statut sérologique du VIH des parents était négatif ou inconnu (ORa = 27,4 [IC 95%: 9,4-80,0]), lorsque l’un ou l’autre ou les deux parents biologiques étaient en vie (ORa = 24,9 [IC 95%: 2,4-250,8]) et lorsque la connaissance du lieu de dépistage était fait par des moyens autres que le professionnel de santé (ORa = 2,9 [IC 95%: 1,0-7,9]).

**Conclusion:**

Notre étude montre une forte prévalence du VIH chez les enfants justifiant la nécessité de réaliser le CDV qui est significativement accepté par leurs parents et tuteurs dans la ville de Lubumbashi.

## Abstract (en)

**Introduction:**

Despite proposals for screening infants or preschool children for HIV infection, the proportion of children who grow or die with unknown HIV status is high in the Democratic Republic of the Congo (DRC). This study aimed to determine the seroprevalence during a voluntary screening and to identify factors associated with Voluntary Counselling and Testing (VCT) for HIV in the paediatric population of non-HIV infected or non-HIV exposed infants and children in Lubumbashi, DRC.

**Methods:**

We conducted a cross-sectional prospective analytical study in 4 community VCT centers divided into 4 health zones in the city of Lubumbashi, DRC (Lubumbashi, Ruashi, Kampemba and Kenya) over the period 1 August 2006 - 31 September 2007. The study aimed to evaluate voluntary testing for HIV among children less than 15 years. The sociodemographic characteristics and the parameters related to Voluntary Counselling and Testing (VCT) for HIV were analyzed. Usual descriptive statistical analyses and logistic regression were perfomed.

**Results:**

Out of 463 children screened for HIV, 41 (8.9%; 95% CI: 6.5%-11.9%) were HIV positive. Voluntary Counselling and Testing (VCT) for HIV in the paediatric population of non-HIV infected or non-HIV exposed infants or children was significantly higher in children over 2 years of age (adjusted odds ratio (AOR)=3.6 [95% CI: 1,1-12,2]) when both of their parents had negative or uknown HIV status (AOR = 27.4 [95% CI: 9,4-80,0]), when either or both of their biological parents were alive (AOR = 24.9 [95% CI: 2,4-250,8]) and when screening programs were not only carried out by health professionals (AOR = 2.9 [95% CI: 1,0-7,9]).

**Conclusion:**

Our study shows a high HIV prevalence among children supporting the need for VCT highly accepted by parents and tutors in the city of Lubumbashi.

## Introduction

En 2011, l’Organisation Mondiale de la Santé (OMS) estimait que 3,3 millions d’enfants de 0 à 14 ans étaient atteints du VIH dans le monde, dont 90% vivaient en Afrique subsaharienne [1]. Beaucoup de nourrissons et d’enfants infectés par le VIH meurent de causes liées au VIH sans que leur statut sérologique VIH soit connu, ou sans avoir reçu des soins adéquats contre le VIH [2]. Comme les adultes, les enfants infectés par le VIH sont pour la plupart diagnostiqués très tardivement dans le cours de la maladie, lorsqu’ils le sont. La progression rapide du VIH chez l’enfant implique que beaucoup meurent, en bas âge ou dans la première enfance, d’affections infantiles courantes ou d’infections opportunistes. Le diagnostic précoce du VIH est donc crucial pour instaurer le traitement antirétroviral aussitôt que possible [3].

En Afrique, et plus particulièrement en République Démocratique du Congo (RDC), les études portant sur le dépistage se sont essentiellement intéressées aux personnes adultes et surtout au dépistage chez la mère dans le cadre de la prévention de la transmission du VIH de la mère à l’enfant (PTME). Le dépistage du VIH chez les enfants a rarement ou peu fait l’objet d’étude singulière. Le dépistage pédiatrique est déjà faisable et acceptable dans les services vaccinaux des milieux à prévalence VIH élevée [4], qui ne couvrent que les 9 premiers mois de vie. La période d’après, n’étant pas couverte par ces services, marque une période cruciale de rattrapage. Malgré le dépistage du VIH proposé au cours des consultations préscolaires qui est non systématique, la proportion des enfants qui croissent ou décèdent sous statut sérologique au VIH inconnu est importante. Selon l’OMS, seulement 8% des nourrissons nés de femmes infectées par le VIH sont soumis à un test virologique dans les 2 premiers mois de vie [2]. Plus des trois quarts des enfants nés de mères infectées par le VIH inscrits pour les soins abandonnent avant 6 mois et près de 85% au 12^ème^ mois du suivi [5, 6]. Le dépistage et le diagnostic de l’infection à VIH sont un enjeu majeur de la lutte contre la maladie, enjeu résidant dans l’instauration précoce d’un traitement antirétroviral, dont l’efficacité sur la réduction de la morbidité et de la mortalité liées au VIH a été clairement démontrée [3]. L’objectif de cette étude était de déterminer la séroprévalence et d’identifier les facteurs associés à l’acceptation du conseil et dépistage du VIH en dehors de la maladie ou de toute exposition au VIH au cours du conseil et dépistage volontaire (CDV) organisé dans une population pédiatrique à Lubumbashi, RDC.

## Méthodes

### Type et cadre d’étude

Il s’agissait d’une étude prospective transversale à visée analytique menée sur la période allant du 1^er^ août 2006 au 31 septembre 2007. L’étude avait été réalisée dans 4 centres communautaires de CDV répartis dans 4 zones de santé (ZS) de la ville de Lubumbashi en RDC (ZS de Lubumbashi, ZS de Ruashi, ZS de Kampemba et ZS de Kenya). Les clients de ces centres venaient de toutes les communes de la ville de Lubumbashi comprenant les résidences urbaines (commune Lubumbashi, commune Kenya et commune Kamalondo) et les résidences urbano-rurales (commune Kampemba, commune Ruashi, commune Katuba et commune Annexe).

Ces 4 centres communautaires de CDV fonctionnaient indépendamment des hôpitaux généraux de référence de ZS où ils étaient implantés. Ils étaient appuyés par l’organisation non gouvernementale (ONG) internationale « Family Health International » (FHI en sigle) et mis en œuvre par « AMOCONGO » une ONG locale. Ces ONGs avaient fourni à ces centres un appui logistique conséquent notamment en intrants (tests de dépistage, suivi biologique, outil de rapportage de données) ; de même, tous les conseillers de ces centres avaient été formés sur la technique de counseling et de dépistage ainsi que sur la récolte et la gestion de données ayant servi au présent travail.

### Population et variables d’étude

Cette étude multicentrique avait ciblé tous les enfants âgés de moins de 15 ans dépistés dans les centres communautaires ci-haut cités. Après le dépistage au VIH, tout enfant séropositif était référé pour un suivi gratuit dans l’hôpital général de référence le plus proche. Ce suivi concernait les consultations, les examens paracliniques (comptage du nombre de lymphocytes CD4, bilan sanguin complet, dépistage de la tuberculose) ainsi que la thérapie antirétrovirale. Les données en rapport avec l’enfant et ses parents avaient été recueillies sur une fiche individuelle préétablie. Les informations recueillies étaient: l’âge et le sexe de l’enfant, la place dans la fratrie, la résidence, la religion, parents vivants ou décédés, le statut sérologique des parents, le moyen de connaissance du lieu de dépistage, la raison évoquée de dépistage, le résultat du test, le stade clinique de l’infection à VIH selon l’OMS (pour les cas positifs).

### Dépistage de l’infection à VIH

**Figure 1:**
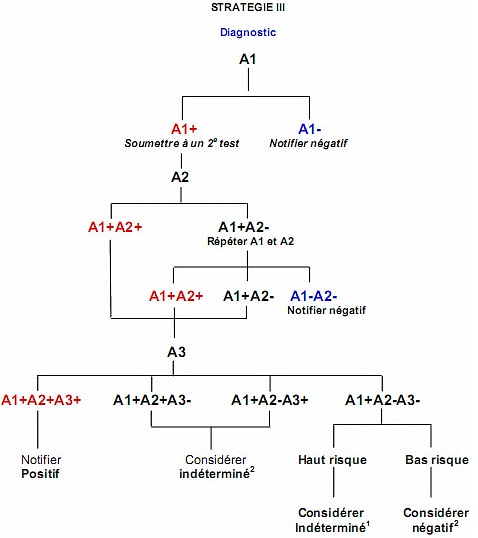
Algorithme du diagnostic rapide du VIH au cours de CDV

Avec les tests rapides couramment utilisés actuellement en RDC, le dépistage de l’infection à VIH chez les enfants se fait à partir de 18 mois. Quand des enfants sont conduits à un site de CDV pour un test de dépistage, le conseiller doit rencontrer les parents ou les tuteurs pour chercher à savoir ce qui motive le dépistage. Le conseiller encourageait les parents/tuteurs à se faire dépister eux aussi. La stratégie utilisée pour le dépistage était la stratégie III proposée par les normes et directives nationales [7]. Cette stratégie prévoyait l’utilisation de trois tests rapides à savoir, Determine™ HIV1/2, Unigold™ HIV et Double-Check™ HIV1&2. Le résumé de la stratégie est repris par la Figure 1 [7].

### Analyses statistiques

Pour l’analyse des données, les statistiques descriptives usuelles ont été utilisées ainsi qu’une mesure d’association (Odds ratio [OR]) entre la raison du dépistage volontaire du VIH comme variable dépendante et les variables indépendantes suivantes: l’âge de l’enfant, le sexe, la position dans la fratrie, la résidence, le statut sérologique au VIH des parents, parents biologiques vivants ou décédés, le moyen de connaissance du lieu de dépistage et la religion des parents. Les facteurs atteignant un degré de signification de p < 0,05ont été retenus comme variables candidates pour l´analyse multi variée. Ils ont été introduits dans un modèle de régression logistique multiple par la méthode d’entrée en bloc. Le seuil de signification était fixé à 5% et les intervalles de confiance à 95% (IC95%). Les analyses ont été réalisées à l´aide des logiciels Epi Info 7.1 et STATA 12.

### Considérations éthiques

Cette étude avait été autorisée par le comité d’éthique de l’Université de Lubumbashi. Les autorités sanitaires ont donné leur accord par écrit. Le consentement libre et éclairé à participer à l’étude a été obtenu verbalement auprès des tuteurs répondants des enfants enrôlés.

## Résultats

Au total, 463 enfants ont été dépistés au cours de la période d’étude. L’âge médian était de 9 ans (extrêmes: 2-14 ans) et 7,3% étaient âgés de 2 ans. Deux cent quarante-quatre (52,7%) étaient de sexe féminin. Les enfants occupant la première position dans la fratrie représentaient 10,8% et ceux habitant dans un milieu urbano-rural 52,5%. S’agissant des caractéristiques des parents, on a constaté que 42,8% étaient de religion pentecôtiste et 6,9% avaient un statut sérologique au VIH positif alors que 30,5% ignoraient leur statut. Trois pourcent d’enfants dans notre série étaient orphelins de deux parents (Tableau 1).

**Tableau 1:** Caractéristiques sociodémographiques de la population étudiée

Variable	Effectif (n=463)	Pourcentage
**Age**		
2 ans	34	7,34
3-5 ans	88	19,01
6-9 ans	121	26,13
10-14 ans	220	47,52
Médiane (extrêmes)	9 ans	(2 – 14 ans)
**Sexe**		
Féminin	244	52,0
Masculin	219	47,30
**Rang dans la fratrie**		
1	50	10,80
2	155	33,47
3	139	30,02
4	64	13,83
≥5	55	11,88
**Résidence**		
Urbano-rurale	243	52,48
Urbaine	220	47,52
**Religion**		
Pentecôtistes	198	42,76
Catholiques	128	27,65
Méthodistes	75	16,20
Musulmans	34	7,34
Autres	28	6,05
**Statut sérologique VIH des parents**		
Positif	32	6,91
Négatif	290	62,63
Inconnu	141	30,46
**Parents en vie**		
Oui	449	96,98
Non	14	3,02

**Figure 2:**
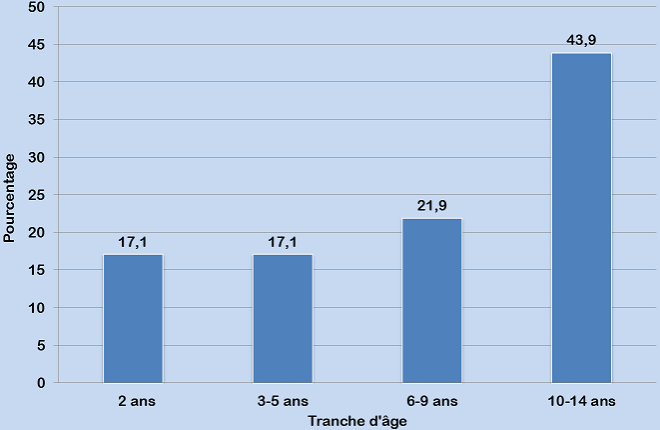
Distribution des enfants testés positifs selon l’âge

**Figure 3:**
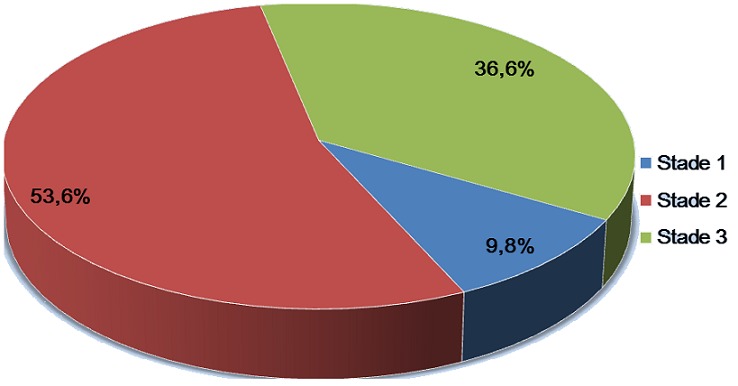
Distribution des enfants testés positifs selon le stade clinique de l’OMS

Le Tableau 2 Montre les paramètres relatifs au CDV. Dans 30,9% des cas (143/463), le moyen de connaissance du lieu de dépistage était un ami de parents ou de tuteurs répondants. La raison évoquée de dépistage était le désir de connaissance du statut sérologique de l’enfant dans 91,6% et le taux de post-testés étaient de 86,6%. Sur les 463 enfants dépistés du VIH, 41 ont été testés positifs, soit une séroprévalence de 8,9% (IC à 95% : 6,5%-11,9%). L’âge médian de ces derniers était de 8 ans (extrêmes: 2-14 ans) et près de 44% étaient dépistés entre 10 et 14 ans (Figure 2). Plus de la moitié (22/41 soit 53,6%) étaient au stade clinique 2 et 36,6% au stade clinique 3 de l’OMS (Figure 3).

**Tableau 2:** Paramètres relatifs au dépistage volontaire

Variable	Effectif (n=463)	Pourcentage
**Moyen de connaissance du lieu de dépistage**		
Ami de parents/tuteurs	143	30,89
Relais communautaires	104	22,46
Agents de santé	79	17,06
Autre client du centre	57	12,31
Eglises	47	10,15
Banderoles	33	7,13
**Raison évoquée de dépistage**		
Désir de connaissance	424	91,58
Maladie	26	5,62
Parents VIH+	7	1,51
Agression sexuelle	3	0,64
Frère/Sœur VIH+	2	0,43
Antécédent de transfusion	1	0,22
**Résultat du test**		
Négatif	422	91,14
Positif	41	8,86
**Post testés**		
Oui	401	86,61
Non	62	13,39

Le Tableau 3 Montre les facteurs associés au désir de connaissance du statut sérologique du VIH de l’enfant en dehors de la maladie ou de toute exposition au VIH. L’acceptation du conseil et dépistage volontaire du VIH en dehors de la maladie ou de toute exposition au VIH était significativement plus élevée lorsque l’enfant était âgé de plus de 2 ans (ORa=3,6 [1,1,12,2]), que le statut sérologique au VIH des parents était négatif ou inconnu (ORa = 27,4 [9,4-80,0]), que l’un ou l’autre ou les deux parents biologiques étaient en vie (ORa=24,9 [2,4-250,8]) et que la connaissance du lieu de dépistage était fait par des moyens autres que le professionnel de santé (ORa = 2,9 [1,0-7,9]).

**Tableau 3:** Facteurs associés au désir de connaissance du statut sérologique du VIH de l’enfant en dehors de la maladie ou de toute exposition au VIH

Variable	Désir de connaissance n (%)	Analyse univariée		Analyse multivariée	
		OR brut [IC95%]	p	OR ajusté [IC95%]	p
**Age**					
2 ans (n=34)	26 (76,5)	1,0	-	1,0	-
>2 ans (n=429)	398 (92,8)	3,9 [1,6-9,5]	0,002	3,6 [1,1-12,2]	0,032
**Sexe**					
Masculin (n=219)	198 (90,4)	1,0	-	-	-
Féminin (n=244)	226 (92,6)	1,3 [0,6-2,5]	0,491	-	-
**Place dans la fratrie**					
1 (n=50)	40 (80,0)	1,0	-	1,0	-
≥2 (n=413)	384 (93,0)	3,3 [1,5-7,2]	0,004	2,8 [0,8-9,5]	0,087
**Statut sérologique au VIH des parents**					
Positif (n=32)	9 (28,1)	1,0	-	1,0	-
Négatif/inconnu (n=431)	415 (96,3)	64,2 [26,4-166,0]	<0,0001	27,4 [9,4-80,0]	<0,0001
**Statut des parents biologiques**					
Décédé (n=14)	1 (7,1)	1,0	-	1,0	-
En vie (n=449)	423 (94,2)	211,5 [26,6-1679,7]	<0,0001	24,9 [2,4-250,8]	0,006
**Résidence**					
Urbano-rurale (n=243)	205 (90,1)	1,0	-	-	-
Urbaine (n=220)	219 (93,2)	1,4 [0,7-2,9]	0,309	-	
Religion des parents					
Pentecôtistes (n=198)	175 (88,4)	1,0	-	1,0	-
Autres (n=265)	249 (93,3)	2,0 [1,0-3,9]	0,048	1,6 [0,6-4,2]	0,278
**Moyen de connaissance du lieu de dépistage**					
Professionnel de santé (n=180)	150 (83,3)	1,0	-	1,0	-
Autres moyens (n=283)	274 (96,8)	6,0 [2,8-13,1]	<0,0001	2,9 [1,0-7,9]	0,035

## Discussion

Cette étude menée dans les centres communautaires avait trouvé une séroprévalence au VIH de 8,9% (IC95%: 6,5-11,9%). Dans une étude menée à Durban (Afrique du Sud), Ramirez-Avila avait rapporté une séroprévalence de 17% (IC95%: 11-25%) [8]. A Harare (Zimbabwe), Bandasona avait trouvé une séroprévalence faible de 2,7% (IC à 95%: 2,2-3,1%) [9]. Notre séroprévalence pédiatrique semble être plus élevée que celle retrouvée dans notre région au sein de la population âgée de 15 à 49 ans qui est de 1,5% [10].

Dans notre série, moins de 10% d’enfants VIH positifs étaient au stade clinique 1 de l’OMS. Ceci illustre le retard de diagnostic dans la population pédiatrique de notre milieu. L’âge médian des enfants infectés par le VIH diagnostiqués était de 8 ans. Cet âge médian est proche de ceux de 7 ans rapporté dans l’étude de Ramirez-Avila et de 9 ans trouvé dans l’étude de Bandasona [8, 9]. Cet âge avancé ne reflète que la situation qui est vécue dans les pays à ressources limitées où plusieurs enfants meurent suite à l’infection à VIH sans que leur statut sérologique VIH ne soit connu ou sans avoir reçu une thérapie antirétrovirale [2]. La plupart d’infections pédiatriques à VIH sont diagnostiqués très tardivement et surtout dans le cours de la maladie. Selon l’UNICEF, l’âge médian auquel le traitement antirétroviral est administré aux enfants séropositifs se situe actuellement entre cinq et neuf ans. Toutefois, lorsque le traitement débute tard, le système immunitaire de l’enfant risque d’être déjà gravement compromis [11].

Le dépistage précoce pédiatrique du VIH ne se fait pas car certains parents/tuteurs ne se fient qu’à la bonne santé apparente de leur enfant et d’autres pensent que les enfants infectés pendant la période périnatale ne survivent pas jusqu´à la fin de l´enfance [12]. De même, les conditions socioculturelles et économiques des accouchées séropositives ainsi que la stigmatisation à la base des taux élevés des perdus de vue dans les stratégies de PTME rapportés par plusieurs auteurs constitueraient un blocage au dépistage précoce [13–16]. Notre étude a révélé que le CDV chez l’enfant en dehors de la maladie ou de toute exposition était acceptable chez les enfants de plus de 2 ans. Ceci pourrait s’expliquer par le fait que, par crainte que leur enfant ne soit dépisté positif, certains parents n’amènent pas les enfants de 2 ans ou moins car ils pensent que les enfants infectés par le VIH en période périnatale n’atteignent pas l’âge de 2 ans [12]. Les connaissances populistes et erronées de l’infection chez l’enfant font adhérer la théorie selon laquelle l’enfant VIH positif ne survit pas au-delà de son deuxième anniversaire [17]. A ceci, s’ajoute la difficulté de confirmation du diagnostic avant 18 mois, soit par absence d’accès à la méthode de réaction en chaîne par polymérase (PCR), soit parce que les résultats reviennent après un délai très long [18]. Nous avons trouvé que les parents/tuteurs qui se reconnaissaient séropositifs amenaient moins leurs enfants au CDV. La divulgation indirecte du statut sérologique parental, couplé à la culpabilité parentale constituent des raisons de ne pas faire dépister les enfants [8, 9, 19]. Selon Davies, les parents séropositifs veulent protéger leurs enfants et eux-mêmes de la discrimination au sein de la famille et la communauté [12].

De même, les enfants dont les parents biologiques étaient en vie étaient plus dépistés que ceux qui étaient orphelins. Le fait que les tuteurs répondants de ces derniers les amenaient moins au CDV pourrait s’expliquer par l’existence d’une discrimination familiale voire communautaire et d’une mauvaise perception autour de l’infection à VIH qui simule une fatalité [17].

Nous avons également trouvé que la connaissance du lieu de dépistage par des moyens autres que le professionnel de santé influençait positivement l’acceptation du CDV chez l’enfant. L’implication de la communauté dans la sensibilisation a favorisé l’accroissement du taux de dépistage volontaire du VIH. Contrairement à nos résultats, à Cotonou (Bénin), Sagbo avait constaté que le dépistage pédiatrique du VIH était mieux accepté par les parents d´enfants en bonne santé apparente lorsqu’il est initié par un personnel de santé [20].

## Conclusion

Notre étude montre une forte prévalence du VIH chez les enfants justifiant la nécessité d’intensifier dans cette population le CDV, qui est accepté par les parents et tuteurs à des enfants dans la ville de Lubumbashi. Il serait bénéfique que cette offre systématique du dépistage fasse partie du paquet de soins de santé au quotidien dans tous nos hôpitaux et établissements de soins de santé.

### Etat des connaissances actuelles sur le sujet

- Le dépistage et le diagnostic de l’infection à VIH sont un enjeu majeur de la lutte contre la maladie, enjeu résidant dans l’instauration précoce d’un traitement antirétroviral;
- Beaucoup de nourrissons et d’enfants infectés par le VIH meurent de causes liées au VIH sans que leur statut sérologique VIH soit connu.

### Contribution de notre étude à la connaissance

- L'étude proposée est la première étude globale dans notre pays, permettant de déterminer la séroprévalence et d’identifier les facteurs associés à l’acceptation du conseil et dépistage du VIH en dehors de la maladie ou de toute exposition au VIH au cours du conseil et dépistage volontaire organisé dans une population pédiatrique à Lubumbashi, République Démocratique du Congo;
- L'étude proposée montre que le dépistage du VIH chez l’enfant fasse partie du paquet de soins de santé au quotidien dans tous nos hôpitaux et établissements de soins de santé.

## Conflits d’intérêts

Les auteurs ne déclarent aucun conflit d'intérêt.
